# Enzymatic Degradation of Polycaprolactone by Cutinase-Producing Bacteria Isolated from Plastic-Contaminated Environments

**DOI:** 10.1007/s12010-026-05709-7

**Published:** 2026-04-25

**Authors:** Sermin Yıldırım, Hamdi Öğüt

**Affiliations:** 1 Department of Biotechnology, Graduate Education Institute, Bursa Technical University, Bursa, 16310 Turkey; 2 Department of Bioengineering, Faculty of Engineering and Natural Sciences, Bursa Technical University, Bursa, 16310 Turkey

**Keywords:** PCL (polycaprolacton), Biodegradation, Tomato cutin, Cutinase

## Abstract

The escalating accumulation of synthetic polymer waste underscores the urgent need for sustainable biodegradation strategies. Among microbial enzymes, cutinases have recently gained prominence for their ability to hydrolyze aliphatic polyesters such as polycaprolactone (PCL). This study reports the isolation and characterization of PCL-degrading bacteria from plastic-contaminated sites in the Bursa region of Turkey, expanding the known ecological distribution of cutinase activity microorganisms. A total of 82 bacterial isolates were obtained, of which 14 exhibited hydrolytic activity on Tween 20, Tween 80, and PCL-containing media, indicative of extracellular esterase/cutinase activity. Three isolates—*Peribacillus sp.* M.2.2, *Peribacillus sp.* K.2.2, and *Stutzerimonas sp.* G.K.5.1—showed the most prominent PCL degradation zones and were selected for further analysis based on 16 S rRNA gene sequencing. Among these isolates, *Stutzerimonas sp.* G.K.5.1 achieved a degradation efficiency of 10.75% in PCL films within 10 days under mesophilic conditions, suggesting measurable enzymatic depolymerization. Optimal parameters for growth, enzyme activity, and total protein content were systematically determined, with tomato-derived cutin identified as an effective natural inducer of cutinase activity. These findings indicate that *Stutzerimonas sp.* G.K.5.1 and *Peribacillus* species possess extracellular cutinases that contribute to PCL depolymerization. The study provides additional insight into bacterial cutinase-mediated polyester degradation and supports the potential use of naturally occurring microorganisms in future enzyme-based plastic bioremediation approaches.

**Supplementary Information:**

The online version contains supplementary material available at 10.1007/s12010-026-05709-7.

## Introduction

The global production of plastics has increased exponentially—from about 2 million metric tons (Mt) in the 1950 s to over 400 Mt in 2023—creating one of the most pressing environmental challenges of the modern era [1]. Plastics such as polyethylene terephthalate (PET), polycaprolactone (PCL), and polybutylene succinate (PBS) are synthetic polymers specifically engineered for durability, strength, and chemical resistance, properties that also render them highly persistent in natural environments. Despite rising public concern and improved recycling infrastructure, only about 9–10% of all plastic waste is effectively recycled, while the remainder is incinerated, landfilled, or dispersed into terrestrial and aquatic ecosystems [2]. This inefficient waste management contributes to greenhouse gas emissions, microplastic formation, and the long-term disruption of ecological processes [3]. Traditional disposal and recycling strategies—such as incineration or mechanical and chemical recycling—are energy-intensive, may release hazardous emissions and degradation products such as microplastics, plastic additives, monomers, and other intermediate compounds that pose potential risks to environmental and human health [4]. In response to these challenges, the European Commission has incorporated sustainable plastic management into its circular economy framework, promoting material reuse, repair, and closed-loop recycling [5]. Achieving this vision requires innovative technologies capable of converting waste polymers into reusable resources without loss of material quality. Enzymatic recycling offers such a route by depolymerizing plastics into their monomeric building blocks, which can then be purified and repolymerized into virgin-quality materials [6]. For instance, industrial-scale processes developed by Carbios utilize PETase and cutinase enzymes to hydrolyze post-consumer PET into terephthalic acid (TPA) and ethylene glycol (EG), enabling closed-loop production of high-quality PET [7]. These examples demonstrate the potential of enzyme-based recycling to transform plastic waste into valuable raw materials under mild, environmentally compatible conditions.

Within this context, enzymatic degradation represents the biological mechanism underlying enzyme-based or enzyme-assisted recycling processes. Enzymes act as biological catalysts that accelerate chemical reactions under moderate temperature and pH, allowing precise control over degradation pathways without generating hazardous residues [8–12]. Among polyester-hydrolyzing enzymes, cutinases (EC 3.1.1.74) are of particular interest due to their ability to cleave ester bonds in both natural and synthetic polyesters. Cutinase, a serine esterase belonging to the α/β-hydrolase family, contains a conserved Ser–His–Asp catalytic triad and can hydrolyze the plant polymer cutin—the structural component of the cuticle—as well as synthetic polyesters such as PET and PCL [13, 14]. Unlike lipases, which require interfacial activation, cutinases act directly on soluble or emulsified substrates [15, 16], making them versatile tools for polyester degradation under laboratory and industrial conditions. Cutinases have been identified in fungi (e.g., *Fusarium solani*, *Humicola insolens*, *Fusarium oxysporum*) [17–19], in bacteria (e.g., *Thermobifida fusca*,* Pseudomonas cepacia*, *Mycobacterium marinum*) [20–22], and even in plants [23]. Several recent studies have highlighted their potential for biotechnological plastic degradation. For instance, enzymes from *Fusarium oxysporum* and *Thermobifida fusca* can depolymerize PET and PCL under ambient conditions [24–26], and genetically engineered cutinase–lipase hybrids have shown improved efficiency on composite plastics [27]. However, most existing research focuses on fungal cutinases, while bacterial cutinases remain comparatively understudied, especially those originating from environmental samples exposed to plastic pollution.

A practical strategy to stimulate cutinase production involves supplementing growth media with plant-derived cutin, a natural polyester composed of cross-linked hydroxy fatty acids. Because its structure closely resembles that of synthetic polyesters, cutin can serve as an effective and inexpensive inducer of cutinase synthesis [28]. Among various plant sources, tomato peel cutin is abundant, renewable, and rich in ω-hydroxy fatty acids, yet its potential as a bacterial cutinase inducer remains underexplored.

Given this background, the present study was designed to: (i) isolate and identify bacterial strains capable of degrading PCL from plastic waste samples collected in Bursa, Turkey; (ii) screen the isolates for hydrolytic activity on Tween 20, Tween 80, and PCL substrates; (iii) identify the most active isolates by 16 S rRNA gene sequencing; (iv) determine optimal conditions for bacterial growth and enzyme production; and (v) evaluate the effect of tomato-derived cutin on extracellular cutinase activity.

By integrating environmental microbiology with enzymatic biodegradation, this study provides new insight into bacterial systems capable of polyester degradation, introduces *Stutzerimonas sp.* G.K.5.1 as a promising cutinase producer, and proposes a novel, cost-effective approach using tomato cutin as a natural inducer for microbial enzyme production. These findings advance the development of sustainable, bio-based strategies for managing synthetic plastic waste.

## Materials and Methods

### Materials

The enzyme substrate 4-nitrophenyl butyrate (p-NPB) was purchased from Medchem (Monmouth Junction, USA). Polycaprolactone (PCL; average Mn ≈ 80,000) was obtained from Sigma-Aldrich (USA), Dimethyl sulfoxide (DMSO) from Chem-Lab (Zedelgem, Belgium), phosphoric acid from Kimyalab (Istanbul, Turkey), methanol from Isolab (Wertheim, Germany), and Triton X-100 from Tekkim (Bursa, Turkey). All chemicals used for media preparation were supplied by Merck (Darmstadt, Germany). Unless otherwise stated, all solutions and reagents were prepared using deionized water.

### Cutin Extraction

Tomatoes obtained from local markets were blanched in boiling water for 2 min to facilitate peel removal. A pH 3.5 oxalate buffer was prepared, and the tomato peels were boiled in this buffer for 30 min. The boiled peels were then filtered, rinsed with distilled water, and dried overnight at 40 °C. The dried peels were subsequently treated with a chloroform–methanol solution (2:1, v/v) at 40 °C for 24 h, followed by another drying step. The residue was then suspended in 50 mM acetate buffer (pH 4.0) containing cellulase (5 g/L) and pectinase (1 g/L), and the mixture was stirred at room temperature for 16–24 h. After enzymatic treatment, the peels were filtered, washed with distilled water, and dried at 40 °C. Finally, the peels were rewashed with chloroform–methanol (2:1, v/v) to remove residual pigments and lipids, then dried and ground into a fine powder, as described previously [29, 30]. To minimize potential variability due to seasonal or source-related differences in tomato composition, all cutin extractions were performed using tomatoes from the same local batch and season. The extracted cutin was stored in airtight containers at room temperature until use. Extracted tomato cutin was used as a natural polyester substrate and inducer to stimulate extracellular cutinase production during bacterial cultivation.

### Sample Collection and Microorganism Isolation

Plastic waste samples were collected from various locations across the Bursa province, including lakeshores, seashores, seabeds, and historical landfill sites. Samples were transferred into sterile polyethylene bags and transported to the laboratory under aseptic conditions. Upon arrival, serial dilutions (ranging from 10⁻¹ to 10⁻⁸) were prepared using sterile saline solution, and aliquots were spread onto Nutrient Agar (NA) plates. Control plates containing only sterile NA were incubated in parallel to monitor possible contamination. All experiments were performed in triplicate. The inoculated plates were incubated at 30 °C for seven days to allow visible colony development. After the incubation period, morphologically distinct colonies were selected and subjected to Gram staining to determine basic cellular and structural characteristics. Selected isolates were preserved at 4 °C for further analysis. For long-term preservation, the isolates were cryopreserved in sterile glycerol (15–30% v/v) and stored at −80 °C.

### Cutinase Production by the Isolated Microorganisms

To determine the cutinase production capacity of the isolated strains, a cutinase selective medium was prepared. A modified version of the method described previously was employed [30, 31]. The medium consisted of 2.4% nutrient agar (NA), 0.4% tomato cutin, and 0.1% phenol red indicator dye, and was incubated at 37 °C for 48 h.

### Screening and Evaluation of Polycaprolactone (PCL) Degradation

A total of 82 bacterial isolates were screened for their ability to degrade PCL. The screening was carried out in two stages: (1) qualitative assessment using PCL-agar plate assays and (2) quantitative evaluation through PCL film weight loss experiments.

### PCL Degradation Assay on Agar Medium

For the preparation of PCL-agar medium, 0.03 g of PCL was dissolved in 6 mL of acetone by stirring at 50 °C for 20 min until fully solubilized. The solution was then mixed thoroughly with 50 mL of warm, pre-prepared Nutrient Agar (NA) and poured into sterile glass Petri dishes [32]. Bacterial isolates were inoculated onto the surface of the solidified medium using sterile swabs via spot inoculation. Plates were then incubated at 30 °C for 72 h. Following incubation, the plates were examined for zones of clearance or discoloration surrounding bacterial colonies, which indicated enzymatic degradation of PCL. The diameters of these zones were measured to assess degradation activity.

### Quantitative Determination of PCL Weight Loss

Isolates that formed degradation zones on PCL-agar were selected for further testing to quantify polymer degradation. For film preparation, 0.3 g of PCL was dissolved in 15 mL of chloroform by heating at 60 °C for 30 min. The solution was poured into Petri dishes and allowed to air dry in a fume hood for 1–2 days, forming thin, uniform PCL films [33]. Dried films were cut into ~ 2 × 2 cm segments weighing 0.04 g each. Prior to incubation, both surfaces of the films were sterilized under UV light for 30 min. Each film was then transferred into 50 mL of Minimal Salt Medium (MSM) and incubated at 30 °C in a shaking incubator at 180 rpm for 10 days. Control samples were maintained without microbial inoculation. Post-incubation, films were recovered, rinsed with distilled water, and dried overnight at 30 °C [34]. Final weights were recorded, and the percentage of weight loss was calculated using the formula:

***W***: weight loss, ***W₀***: initial weight, ***W₁***: final weight

$$W\;\left(\%\right)\;=\frac{\left(W_o-W_1\right)}{\left(W_o\right)}\;\times\;100$$

### Isolate Identification

The selected bacterial isolates were taxonomically identified via 16 S rRNA gene sequencing, performed by BMLabosis (Ankara, Turkey). Genomic DNA was extracted, and the 16 S rRNA gene was amplified using universal primers: 27 F (5′-AGAGTTTGATCMTGGCTCAG-3′) and 1492R (5′-TACGGYTACCTTGTTACGACTT-3′). The PCR protocol consisted of an initial denaturation at 95 °C for 5 min, followed by 30 amplification cycles comprising: denaturation at 95 °C for 30 s, annealing at 57 °C for 30 s, and extension at 72 °C for 90 s. A final elongation step was performed at 72 °C for 5 min. The PCR products were separated by 1% agarose gel electrophoresis, purified, and sequenced. Sequenced products were compared with those available in the NCBI GenBank database using the Basic Local Alignment Search Tool (BLASTn) against the nucleotide collection (nt) database. Species-level similarity was further confirmed through the EzBioCloud platform. Phylogenetic trees were generated using the Neighbor-Joining method in MEGA version 11 with 1000 bootstrap replicates to assess branch support.

### Scanning Electron Microscopy (SEM)

The surface morphology of PCL films was analyzed before and after bacterial treatment using scanning electron microscopy (SEM). Imaging was performed with a Zeiss Gemini 300 microscope equipped with an EDS detector (Bruker XFlash 6I100). Analyses were carried out at MERLAB, Central Research Laboratory, Bursa Technical University, Turkey. Control PCL films were processed and imaged under identical SEM conditions to ensure that observed surface features in treated films reflect enzymatic degradation rather than sample preparation artifacts.

### FTIR Analysis

Fourier Transform Infrared (FTIR) spectroscopy was performed using a Thermo Scientific Nicolet iS50 spectrometer at the MERLAB of Bursa Technical University. Spectra were recorded in the range of 4000–600 cm⁻¹ with a resolution of 4 cm⁻¹.

### Screening for Polyesterase Activity Using Tween 20 and Tween 80

To evaluate extracellular polyesterase activity, the ability of isolates to hydrolyze Tween 20 and Tween 80 was assessed. These nonionic surfactants serve as model substrates for esterases and lipases. Upon enzymatic hydrolysis, the oleic acid released reacts with Ca²⁺ ions in the medium to form an opaque calcium precipitate, visible as a turbid zone surrounding colonies with lipolytic activity. The screening medium contained the following components per liter: 10 g peptone, 5 g NaCl, 0.1 g CaCl₂, 20 g agar, and 10 mL of either Tween 20 or Tween 80. The pH was adjusted to neutrality before autoclaving. Sterile Petri dishes were poured with the medium, and isolates were inoculated by spot application using sterile micropipette tips. Plates were incubated at 30 °C for 5–7 days. The formation of opaque zones around the colonies was interpreted as positive for lipase/esterase activity [35]. It should be noted that Tween hydrolysis indicates general extracellular esterase/lipase activity, which may not exclusively reflect cutinase activity. Triplicate experiments and negative controls (uninoculated medium) were used.

### Screening of Cutinase Activity

Bacterial isolates were cultivated in 20 mL of Minimal Salt Medium (MSM) supplemented with 0.2% (w/v) cutin as the sole carbon source. The composition of MSM was as follows: 1.0 g/L K₂HPO₄, 0.2 g/L KH₂PO₄, 0.1 g/L NaCl, 0.002 g/L CaCl₂·2 H₂O, 0.1 g/L (NH₄)₂SO₄, 0.5 g/L MgSO₄·7 H₂O, and 0.1 g/L FeSO₄·7 H₂O. Cultures were grown in 100 mL Erlenmeyer flasks at 30 °C with shaking at 150 rpm for 7 days [36–38]. Every 24 h, 1.5 mL aliquots of the culture were collected and centrifuged at 10,000 × g for 20 min. The supernatants were used for the subsequent analysis of cutinase activity.

### Cutinase Activity Assay

Cutinase activity was determined spectrophotometrically using p-nitrophenyl butyrate (p-NPB) as the substrate. The reaction mixture (1.0 mL total volume) contained 12 mM p-NPB, 50 mM phosphate buffer (pH 7.0), 100 µL Triton X-100, and 100 µL of enzyme-containing culture supernatant. Prior to enzyme addition, the mixture was incubated at 37 °C for 1 min to equilibrate. The reaction was initiated by adding the enzyme, and the release of p-nitrophenol was monitored by measuring absorbance at 410 nm [20, 39]. One unit (U) of cutinase activity was defined as the amount of enzyme that releases 1 µmol of p-nitrophenol per minute under assay conditions. Cutinase/esterase activity was expressed as U/mL of culture supernatant, and increases in volumetric activity reflect total enzyme per culture volume rather than per-cell induction.

### Total Protein Content by Bradford Method

The total protein concentration in the culture supernatants was quantified using the Bradford method [40, 41]. The Bradford reagent was prepared by dissolving 100 mg of Coomassie Brilliant Blue G-250 in 50 mL of 95% ethanol. Subsequently, 100 mL of 85% phosphoric acid was added, and the final volume was adjusted to 1,000 mL with distilled water. The solution was stirred overnight in the dark and then filtered to remove particulates. The reagent was stored in the dark at 4 °C in bottles. Bovine Serum Albumin (BSA) was used to generate a standard calibration curve. A 0.1 mg/mL stock solution of BSA was prepared, and serial dilutions were made to construct the standard. To each sample or standard, Bradford reagent was added and incubated at room temperature for 5 min. Absorbance was measured at 595 nm using a spectrophotometer. For spectrophotometric protein determination, 50 µL of cell-free culture medium was mixed with 200 µL of Bradford reagent (1:4 ratio) in a 96-well plate. The assay was performed in triplicate. Protein concentrations were determined by comparing sample absorbances to the BSA standard curve [29]. Total protein concentrations of enzyme samples obtained under different production conditions were determined using the Bradford method, with bovine serum albumin as the standard.

### SDS-PAGE Analysis

The molecular weight of the enzyme was estimated by sodium dodecyl sulfate–polyacrylamide gel electrophoresis (SDS-PAGE) [42]. Equal volumes of the culture supernatant (20 µL) were mixed with 5 µL of 4× Laemmli sample buffer (Bio-Rad) and heated at 95 °C for 5 min prior to electrophoresis. The samples were then loaded onto a 12% Tris–glycine polyacrylamide gel. A pre-stained protein molecular weight marker (5 µL; Thermo Scientific) was used as a reference. Electrophoresis was performed at 80 V for 1 h followed by 90 V for 1 h 25 min. After electrophoresis, the gel was stained with Coomassie Brilliant Blue G-250 for 1 h at room temperature with gentle agitation in the dark. Excess stain was removed using distilled water, and the gel image was captured using a ChemiDoc imaging system (Thermo Scientific).

### Effect of Temperature, pH, and Cutin Concentration on Cutinase Activity

To evaluate the influence of environmental parameters on cutinase activity, bacterial cultures were incubated under varying conditions of temperature, pH, and substrate concentration. For temperature optimization, 100 mL of MSM medium supplemented with 0.2% (w/v) cutin was inoculated with 2% (v/v) of the bacterial culture. The cultures were incubated at four different temperatures (25, 30, 40, and 50 °C) in a shaking incubator at 150 rpm for 7 days. To assess the effect of pH, MSM media were adjusted to pH values ranging from 5.0 to 10.0 (in increments of 1.0) using appropriate buffer systems (acetate for pH 5–6, phosphate for pH 7, and Tris-HCl for pH 8–10). Each flask was inoculated with 2% (v/v) of the microbial suspension and incubated at 30 °C under the same shaking conditions. For the effect of cutin concentration, MSM media were prepared with varying concentrations of cutin (0.2%, 0.4%, 0.6%, 0.8%, 1.0%, 1.5%, and 2.0% w/v). The cultures were inoculated and incubated under identical conditions (30 °C, 150 rpm). At the end of the incubation period, 1.5 mL aliquots from each culture were harvested and centrifuged at 10,000 × g for 20 min. The supernatants were collected for the determination of cutinase activity (U/mL) and total protein content(mg).

### Statistical Analysis

All experimental data were statistically analyzed using R Studio (Version 4.4.2). For datasets following a normal distribution (verified via Shapiro-Wilk test), differences among groups were assessed using one-way Analysis of Variance (ANOVA). Post hoc analysis was performed using Tukey’s HSD test when applicable. For the non-normally distributed data, the Kruskal-Wallis test was employed to assess statistical significance. A p-value < 0.05 was considered statistically significant in all tests. The normality assumption was evaluated with the Shapiro-Wilk test on the residuals of each experimental group, and the homogeneity of variances (homoscedasticity) was evaluated with the Levene test. The error bars are expressed as mean ± standard deviation.

## Results

### Preliminary Characterization of Bacterial Isolates and Cutin Preparation

**Fig. 1:**
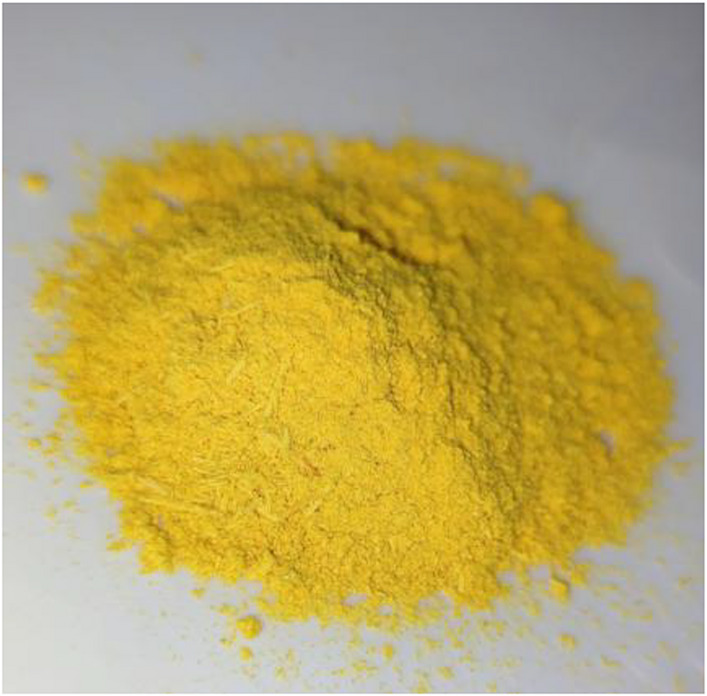
Cutin powder extracted from tomato skins

A total of 82 bacterial strains were isolated from the collected plastic waste samples. These isolates were subsequently screened for their ability to degrade polycaprolactone (PCL) and to produce extracellular cutinase enzymes. Initial screening on PCL-agar plates revealed that 25 of the isolates formed prominent degradation zones. The colony pigmentation of these strains varied and included cream, light cream, pink, yellow, and reddish tones, and was observed on nutrient agar (NA) without the addition of any dyes. Morphologically, the colonies exhibited diverse forms, including rod-shaped, round, and chain-like structures. Gram staining analysis revealed that 21 isolates were Gram-positive and 4 were Gram-negative (Table 1). Cutin used in the enzymatic screening assays was extracted from tomato peels as described. The final product was a yellowish-orange powder, indicating the presence of hydrophobic, polyhydroxy fatty acid derivatives typical of natural cutin (Fig. 1).

**Table 1:** Morphologies of isolates that formed zones on PCL agar

Isolate	NA-Colony colour	NA-Colony shape	Gram (+)/(-)	Tween 20 *AI	Tween 80 *AI	PCL Agar *AI
I.3.1.1	Creamy	Circular	+	6/6 (1.0)	-	5/10 (2.5)
I.4.1	Light Creamy	Circular	+	9/8 (1.12)	11/10 (1.1)	10/6 (1.6)
I.6	Creamy	Rod	+	7/6 (1.16)	-	9/6 (1.5)
IN.3.3	Creamy	Circular	-	12/11 (1.09)	-	16/7 (2.28)
IN.6.2	Creamy	Circular	+	11/11 (1.0)	8/7 (1.14)	14/10 (1.4)
IN.7.1	Pink-Red	Rod	-	-	-	25/12 (2.08)
K.2.2	Creamy	Rod	+	20/18 (1.11)	4/3 (1.33)	30/10 (3.0)
K.2.3	Creamy	Long Rod	+	19/18 (1.05)	-	12/5 (2.4)
B.1.1	Creamy	Circular	+	18/17 (1.05)	22/21 (1.04)	21/9 (2.33)
B.1.2	Creamy	Circular	+	25/22 (1.13)	30/25 (1.2)	30/11 (2.72)
S.1.1	Creamy	Rod	+	19/18 (1.05)	-	12/5 (2.4)
S.1.2	Creamy	Rod	+	13/12 (1.08)	13/12 (1.08)	10/6 (1.66)
S.3.4	Creamy	Circular	-	13/13 (1.0)	-	14/5 (2.8)
G.4	Creamy	Rod	+	20/17 (1.17)	30/24 (1.25)	13/9 (1.44)
M.1.3	Creamy	Rod	+	10/9 (1.11)	16/15 (1.06)	11/7 (1.57)
M.2.2	Clear Pink	Rod	+	15/14 (1.07)	17/16 (1.06)	22/8 (2.75)
M.3.1	Creamy	Circular	+	22/19 (1.15)	18/17 (1.05)	20/10 (2.0)
M.7.2	Clear Creamy	Circular	+	-	-	12/3 (4.0)
M.7.3	Yellow	Circular	+	-	-	20/3 (6.66)
M.8.1	Creamy	Circular	+	10/10 (1.0)	10/9 (1.11)	16/12 (1.33)
M.8.4	Creamy	Circular	+	12/12 (1.0)	12/11 (1.09)	13/10 (1.3)
M.8.5	Creamy	Circular	+	13/12 (1.08)	13/12 (1.08)	10/6 (1.66)
G.K.1	Creamy	Circular	+	10/10 (1.0)	-	8/4 (2.0)
G.K.4	Creamy	Circular	+	20/20 (1.0)	10/10 (1.0)	12/6 (2.0)
G.K.5.1	Clear Creamy	Rod	-	20/18 (1.11)	3/3 (1.0)	16/2 (8.0)

*PCL* Polycaprolacton, *NA* Nutrient Agar, *AI(Activity inhibition) = (hydrolyze zone diameter(mm))/(colony diameter(mm)). Isolates with a high *AI ratio were selected from a population of isolates demonstrating Tween 20, Tween 80, and PCL activity

**Fig. 2:**
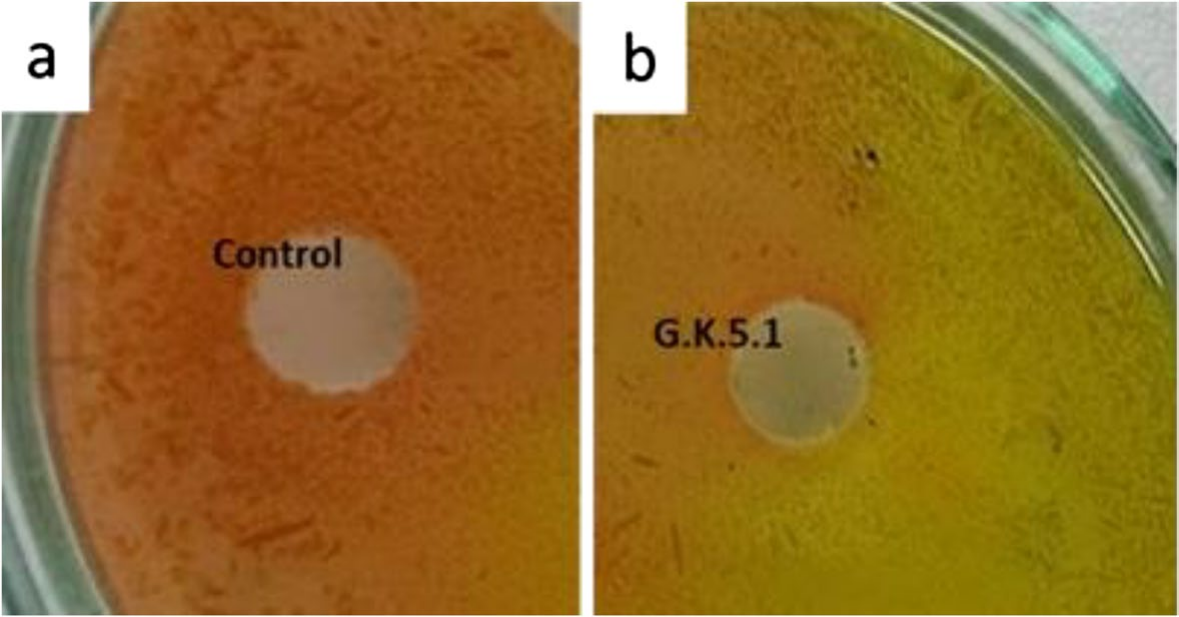
Cutinase activity on screening medium: **a** Negative bacterial control showing no cutinolytic activity, **b** Yellow halo formation in G.K.5.1 isolate showing cutinolytic activity

The isolates obtained from plastic waste were screened for cutinase secretion by inoculating them onto a cutinase selective medium. Cutinase-positive strains were identified by the formation of a yellow halo around the colonies, resulting from the hydrolysis of cutin and the subsequent release of free fatty acids. The liberated fatty acids lowered the pH of the surrounding medium, which was indicated by a color change of the phenol red indicator from dark pink to yellow (Fig. 2).

### Screening of PCL Degradation in Bacterial Isolates

**Fig. 3:**
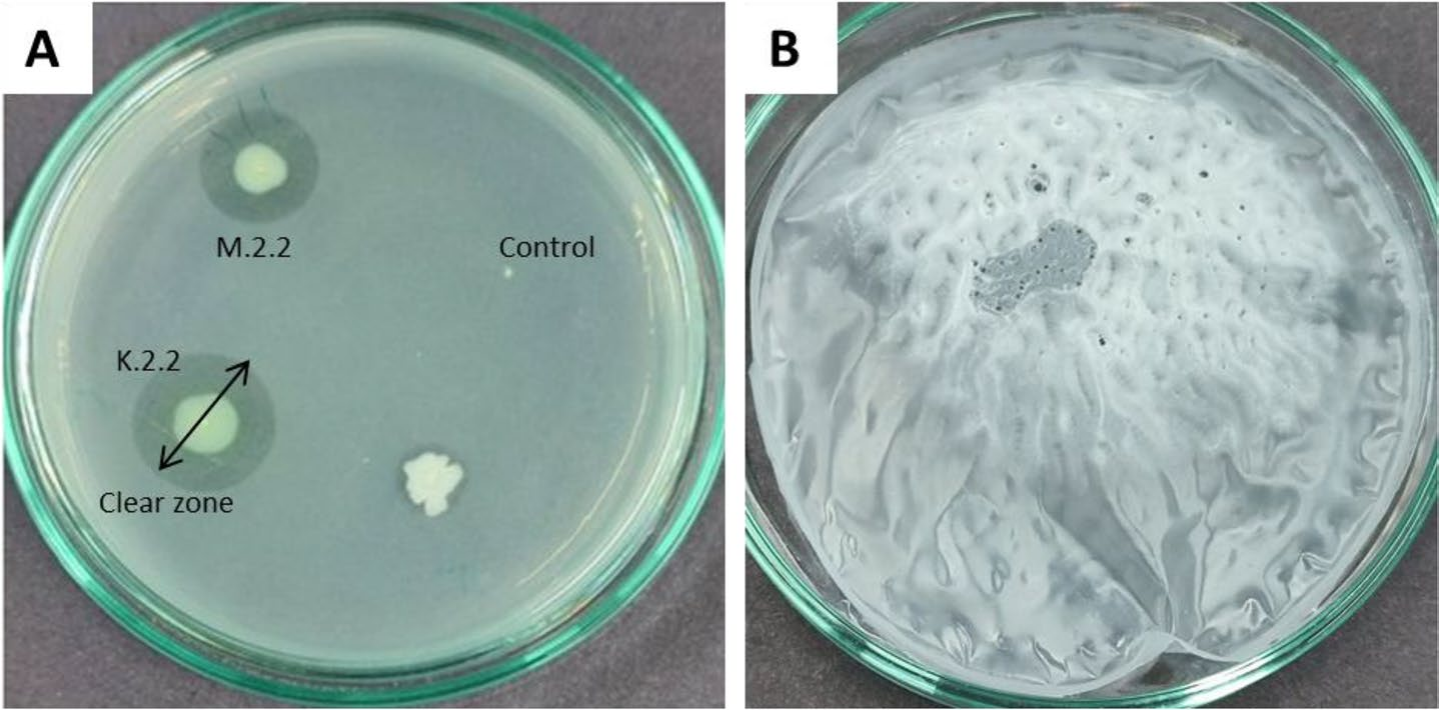
**a** Formation of clear zones on PCL-agar plates M.2.2 (*Peribacillus sp.* M.2.2), K.2.2 (*Peribacillus sp* K.2.2). Control (non-zone forming bacteria); **b** PCL films developed for use in weight loss experiments

**Fig. 4:**
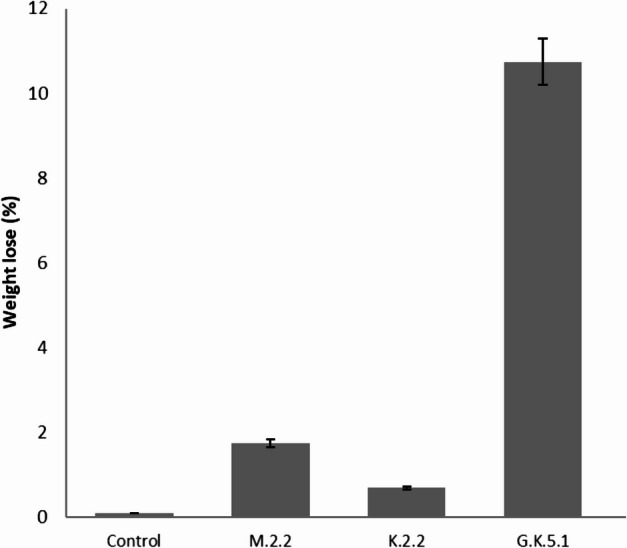
PCL weight loss (%) after incubation with Control, M.2.2 (*Peribacillus sp.* M.2.2), K.2.2 (*Peribacillus sp* K.2.2), and G.K.5.1 (*Stutzerimonas sp.* G.K.5.1) isolates and control. The bars presents the mean values together with their standard deviations (*n* = 3)

Out of the 82 bacterial strains initially screened, 32 isolates demonstrated the ability to degrade polycaprolactone (PCL) on solid media. Among these, 14 isolates exhibited concurrent hydrolytic activity toward both PCL and nonionic surfactants Tween 20 and Tween 80, indicating potential polyesterase or lipase activity. Based on the preliminary screening results, three promising isolates—designated M.2.2, K.2.2 and G.K.5.1 —were selected for quantitative PCL degradation analysis using the weight loss method in Minimal Salt Medium (MSM). PCL films used in this assay were prepared as described (Fig. 3). Following 10 days of incubation at 30 °C with shaking, weight loss of the PCL films was measured. A negative control (medium without bacterial inoculum) was included, showing no detectable degradation. The degradation percentages for isolates M.2.2 (*Peribacillus sp.* M.2.2), K.2.2 (*Peribacillus sp.* K.2.2), and G.K.5.1 (*Stutzerimonas sp.* G.K.5.1) were found to be 1.75%, 0.70%, and 10.75%, respectively (Fig. 4). Among these, G.K.5.1 exhibited the highest degradation capacity, confirming that the observed weight loss is due to bacterial enzymatic activity against PCL polymers. No measurable weight loss was observed in sterile MSM containing polycaprolactone (PCL) without microbial inoculation after 10 days of incubation under standard conditions.

### Scanning Electron Microscopy of PCL Film

**Fig. 5:**
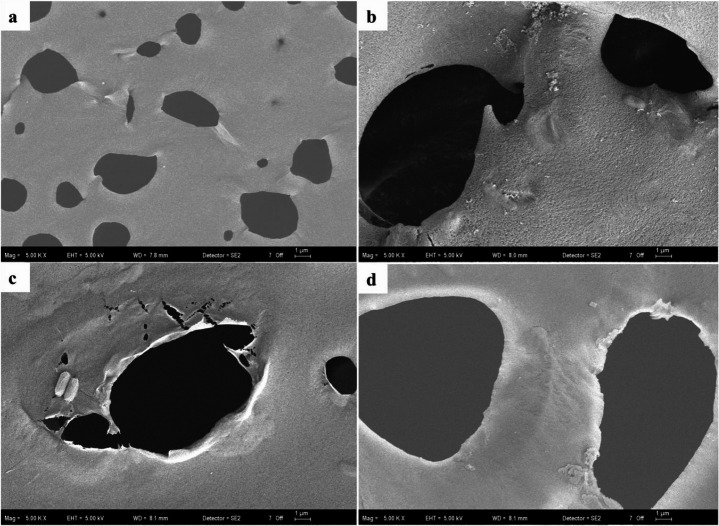
Scanning electron micrographs of PCL films after 10 days of incubation: **a** Control, **b** M.2.2 (*Peribacillus sp.* M.2.2), **c** K.2.2 (*Peribacillus sp* K.2.2), **d** G.K.5.1 (*Stutzerimonas sp.* G.K.5.1) (scale bar: 1 μm; magnification: ×5000)

SEM analysis revealed distinct surface alterations on PCL films following bacterial treatment compared to the untreated control (Fig. 5). After 10 days of incubation with isolates M.2.2, K.2.2 and G.K.5.1, the treated films exhibited noticeable morphological changes, including the presence of surface cracks, holes, and increased pore size. In contrast, control PCL films showed relatively smooth surfaces with only minor irregularities. The surface disruptions observed in the treated samples were more extensive and pronounced than those detected in the control films, indicating substantial physical modification of the polymer surface following bacterial exposure.

### FTIR Analysis

**Fig. 6:**
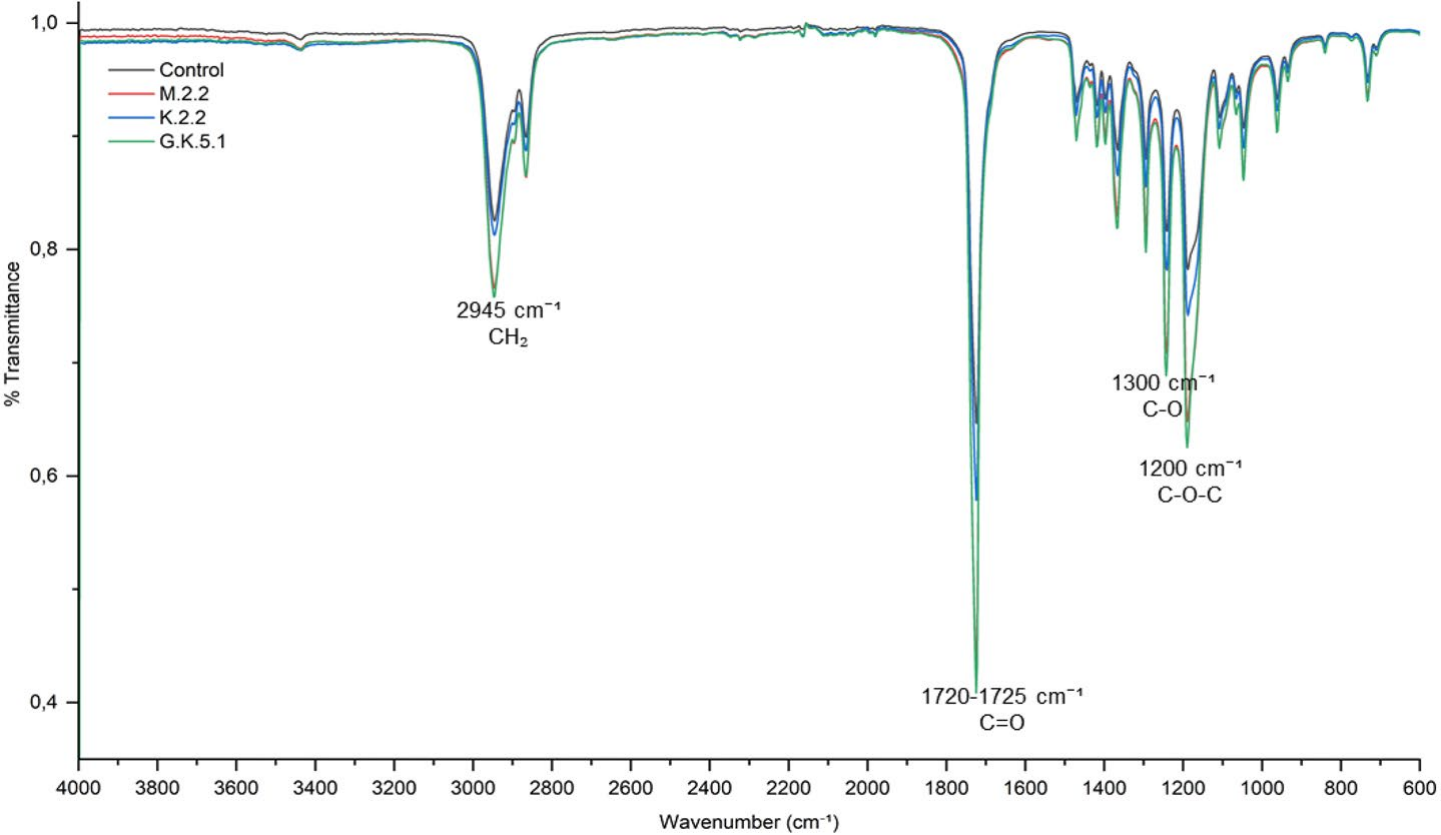
Comparative FTIR analysis of control and microbially treated PCL films. The spectra represent: Control (bacteria-free sample), K.2.2 (*Peribacillus sp.* K.2.2), G.K.5.1 (*Stutzerimonas sp.* G.K.5.1) and M.2.2 (*Peribacillus sp.* M.2.2) samples

FTIR spectra of control and bacteria-treated (*Peribacillus sp*. M.2.2, *Peribacillus sp*. K.2.2, and *Stutzerimonas sp.* G.K.5.1). PCL films are presented in Fig. 6. The characteristic absorption bands of PCL were preserved in all samples. The prominent carbonyl (C = O) stretching band at ~ 1720 cm⁻¹ and the C–O–C stretching vibrations in the 1100–1250 cm⁻¹ region were clearly observed in both control and treated groups. No significant peak shift or new band formation was detected after 10 days of bacterial incubation. However, an increase in the intensity of the carbonyl band at ~ 1720 cm⁻¹ was observed in bacteria-treated samples compared to the control.

### Molecular Identification of Selected Isolates

**Fig. 7:**
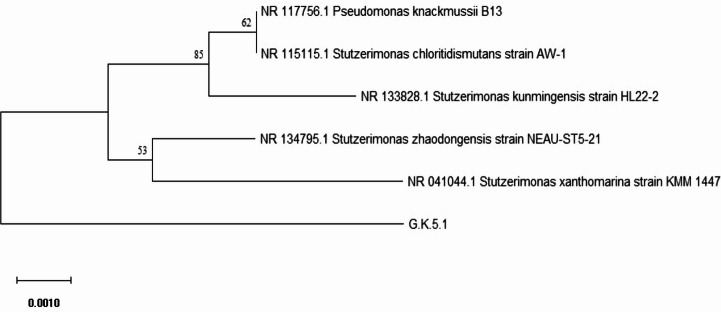
Neighbor-Joining phylogenetic tree based on 16 S rRNA gene sequences showing the strain *Stutzerimonas sp.* G.K.5.1

**Fig. 8:**
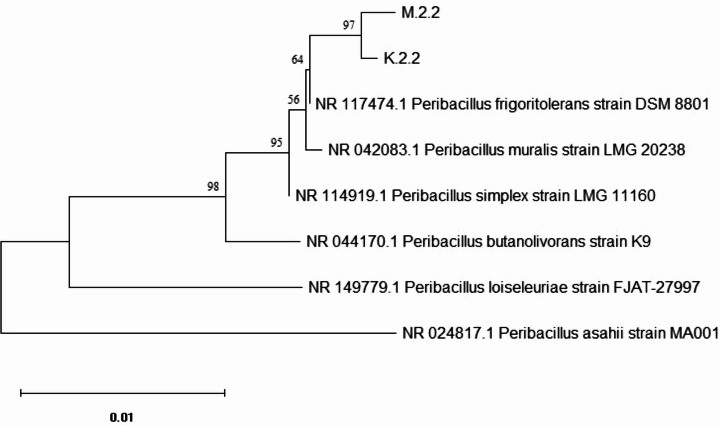
Neighbor-Joining phylogenetic trees based on 16 S rRNA gene sequences showing the strains *Peribacillus sp*. M.2.2 and *Peribacillus sp*. K.2.2

The 16 S rRNA gene sequences of the selected isolates were analyzed to determine their phylogenetic affiliations. Sequence comparisons using BLAST and EzBioCloud revealed that strain G.K.5.1 showed the highest similarity (98.76%) to the type strain of *Stutzerimonas zhaodongensis* (Fig. 7). Phylogenetic analysis placed G.K.5.1 within the genus *Stutzerimonas*, forming a lineage closely related to *S. zhaodongensis*. Accordingly, the strain was affiliated with the genus *Stutzerimonas* and designated as *Stutzerimonas sp*. G.K.5.1. Isolates M.2.2 and K.2.2 exhibited the highest 16 S rRNA gene sequence similarities of 99.59% and 99.64%, respectively, to *Peribacillus frigoritolerans*. In the phylogenetic tree, both strains clustered within the *Peribacillus* clade and formed a well-supported subcluster (bootstrap value: 97%) closely related to *P. frigoritolerans* (Fig. 8). Based on sequence similarity and phylogenetic clustering, these isolates were affiliated with the genus *Peribacillus* and designated as *Peribacillus sp.* strains M.2.2 and K.2.2. Overall, the isolates were consistently classified into their respective genera based on their phylogenetic placement and 16 S rRNA gene sequence analysis.

### Screening of Cutinase Activity

#### Effect of Incubation Time on Cutinase Activity

**Fig. 9:**
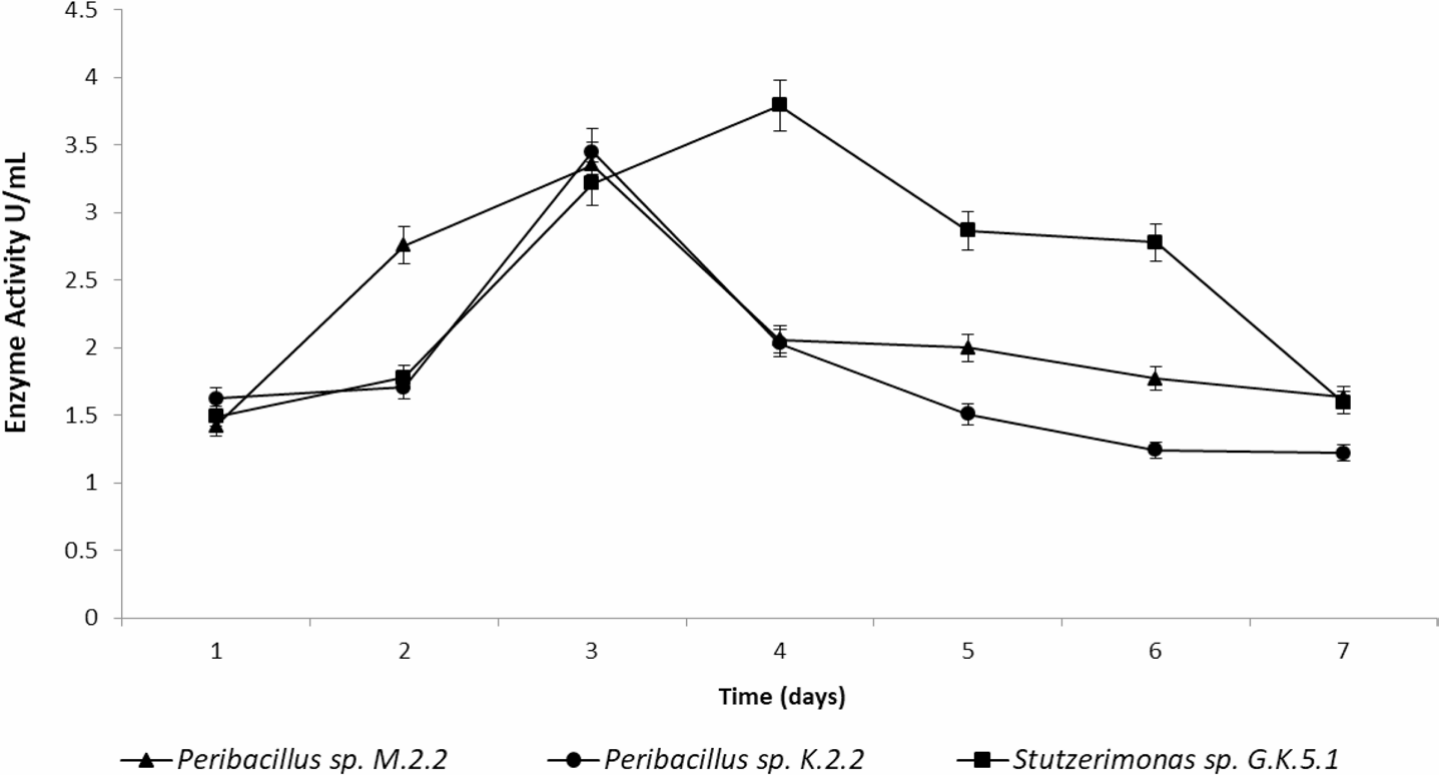
Effect of incubation time on cutinase activity of the bacterial isolates. Statistical analysis was performed using one-way ANOVA followed by Tukey’s test (*P* < 0.05; *n* = 3)

Cutinase activity was evaluated under defined culture conditions in Minimal Salt Medium (MSM) supplemented with 0.2% (w/v) tomato-derived cutin as the substrate, at pH 7.0 and 30 °C. The selected bacterial isolates were incubated with shaking at 150 rpm for 7 days, and cutinase activity was measured daily from the culture supernatants to assess time-dependent enzyme activity. Among the isolates tested, *Peribacillus sp.* M.2.2 exhibited a maximum cutinase activity of 3.35 ± 0.02 U/mL on day 3, while *Peribacillus sp.* K.2.2 reached a peak activity of 3.14 ± 0.002 U/mL also on day 3. In contrast, *Stutzerimonas sp.* G.K.5.1 showed its highest cutinase activity on day 4, reaching 3.80 ± 0.01 U/mL (Fig. 9). For comparative purposes, an additional experiment was conducted using isolate 180, identified as *Fusarium oxysporum*, which was incubated in MSM supplemented with 0.4% (w/v) cutin for 14 days at 30 °C with agitation at 100 rpm. Under these conditions, the maximum cutinase activity reached approximately 1.2 U/mL on day 12, in agreement with previously reported data [36].

Total extracellular protein concentrations in the culture supernatants of selected isolates were quantified using the Bradford assay, with bovine serum albumin (BSA) as the standard. Absorbance was measured at 595 nm, and protein concentrations were calculated from a standard calibration curve (R² = 0.9872). Under these conditions, *Stutzerimonas sp.* GK51 exhibited the highest total protein concentration at 0.19 mg/mL, followed by *Peribacillus sp.* M.2.2 and *Peribacillus sp.* K.2.2, both at 0.12 mg/mL.

**Fig. 10:**
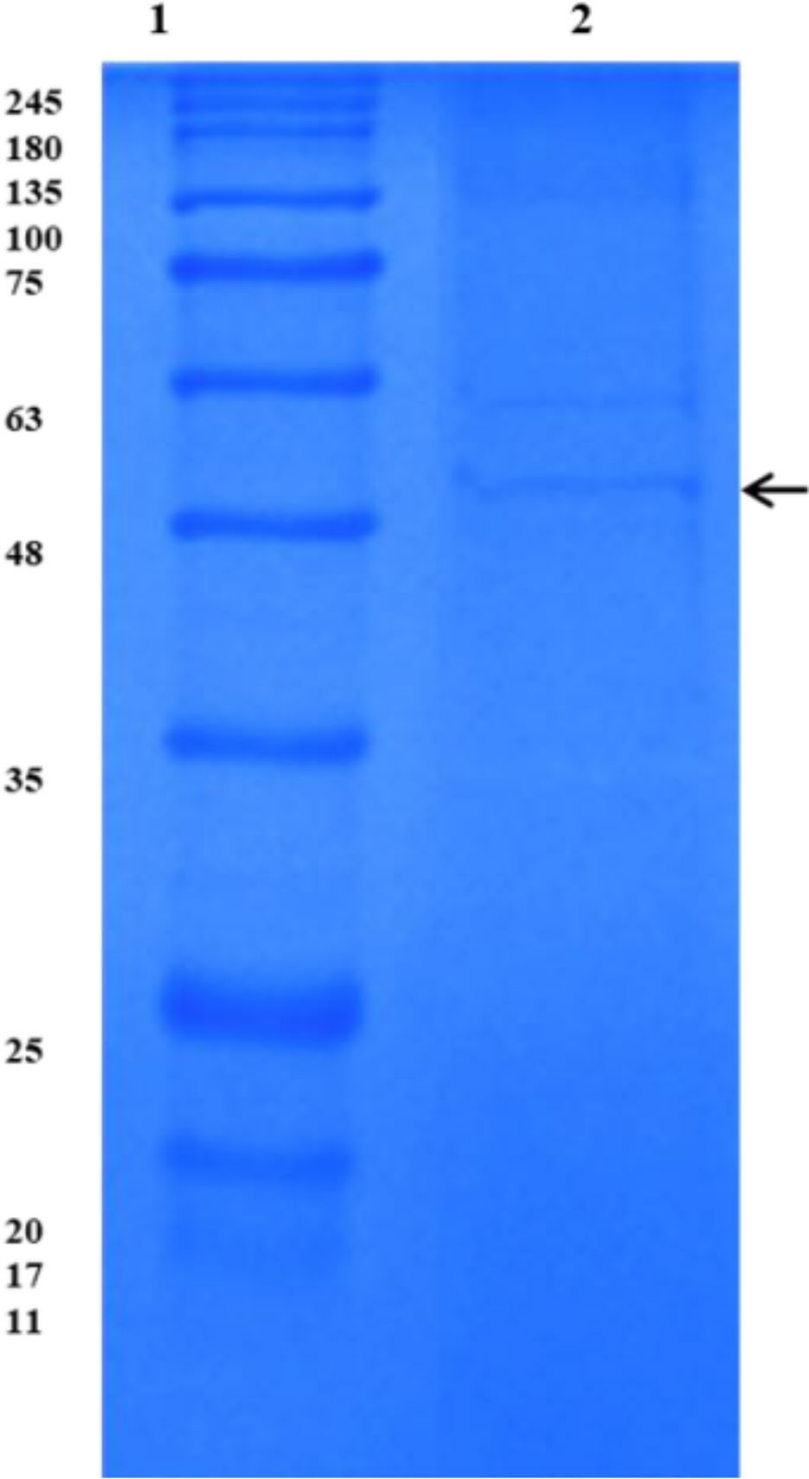
SDS-PAGE analysis of cutinase produced by *Stutzerimonas sp.* G.K.5.1. Lane 1: molecular weight marker (kDa); Lane 2: culture supernatant of *Stutzerimonas sp.* G.K.5.1

To confirm cutinase production at the protein level, SDS-PAGE analysis was performed using the culture supernatant of *Stutzerimonas sp.* G.K.5.1 collected on the day of maximum enzyme activity (day 4). Equal amounts of protein were loaded onto a 12% polyacrylamide gel under denaturing conditions and visualized by Coomassie Brilliant Blue staining. A distinct protein band with an approximate molecular weight of 49 kDa was observed (Fig. 10), which is consistent with the reported molecular weight range of microbial cutinases.

### Effect of Temperature, pH, and Cutin Concentration on Cutinase Activity

#### Effect of Temperature on Cutinase Activity

**Fig. 11:**
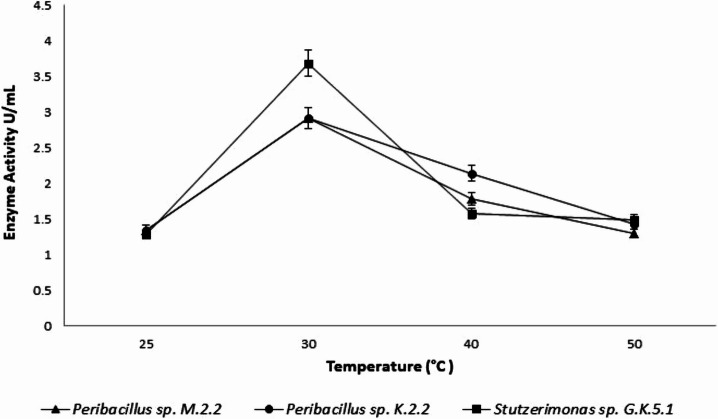
Effect of temperature on cutinase activity of the bacterial isolates. Statistical analysis was performed using one-way ANOVA followed by Tukey’s test (*P* < 0.05; *n* = 3)

To determine the optimal temperature for cutinase activity, the selected isolates were cultured in MSM medium supplemented with 0.2% cutin and incubated at various temperatures (25–50 °C) for 7 days with shaking at 150 rpm. Maximum enzyme activity for all isolates was recorded at 30 °C: *Peribacillus sp.* M.2.2: 2.90 U/mL (p = *0.0216*), *Peribacillus sp.* K.2.2: 2.91 U/mL (p = *0.0015*), *Stutzerimonas sp.* G.K.5.1: 3.67 U/mL (p = *0.0001*) (Fig. 11). Corresponding total protein concentrations at this temperature were 0.11 mg/mL for *Peribacillus sp.* M.2.2, 0.11 mg/mL for *Peribacillus sp.* K.2.2 and 0.19 mg/mL for *Stutzerimonas sp.* G.K.5.1. These findings suggest that 30 °C is the optimal temperature for cutinase activity in these isolates. These results are in alignment with prior studies. For instance, cutinase activities ranging from 11.7 to 15.5 U/mL were reported for *Fusarium oxysporum* after 72 h of incubation at 28–30 °C [43]. *Pseudomonas aeruginosa* strains K799 and DAR41352 produced maximum cutinase similarly at sub-optimal growth temperatures below their optimum (37 °C) [44].

#### Effect of pH on Cutinase Activity

**Fig. 12:**
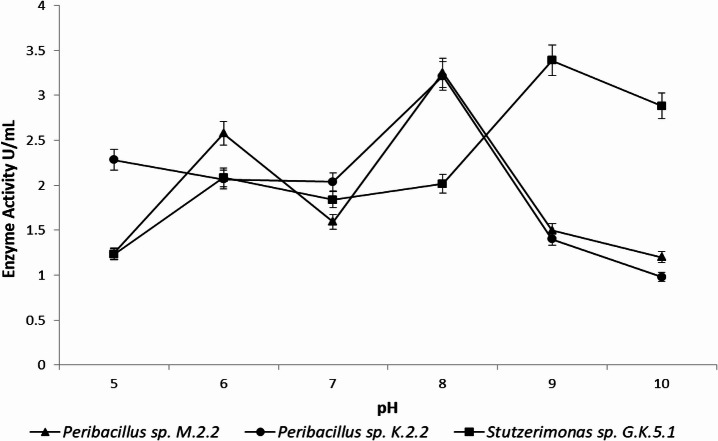
Effect of pH on cutinase activity of the bacterial isolates. Statistical analyses were performed using one-way ANOVA followed by Tukey’s test for normally distributed data and Kruskal–Wallis followed by Dunn’s test for non-normally distributed data (*P* < 0.05; *n* = 3)

To investigate the influence of pH on cutinase activity, the selected bacterial strains were inoculated into MSM medium adjusted to different initial pH values (ranging from 5.0 to 10.0) and incubated at 30 °C with agitation at 150 rpm for 7 days. Cutinase activity was quantified from culture supernatants. Isolate *Peribacillus sp.* M.2.2 exhibited the highest enzyme activity at pH 8.0, with a value of 3.25 U/mL (p = *0.0033*). Isolate *Peribacillus sp.* K.2.2 showed peak activity of 3.21 U/mL at pH 8.0 (p = *0.0167*). Isolate *Stutzerimonas sp.* G.K.5.1 demonstrated the highest activity at pH 9.0, with a measured value of 3.38 U/mL (p = *0.0507*) (Fig. 12). These results indicate that alkaline conditions favor optimal cutinase activity in the studied isolates, with a shift toward higher pH for isolate *Stutzerimonas sp.* G.K.5.1. Comparable findings have been reported in the literature. For example, optimal cutinase activity at neutral pH (pH 7.0) has been reported for *Bacillus sp*. KY0701 grown in MSM medium across a similar pH range, supporting the pH-dependent nature of cutinase activity [34].

#### Effect of Cutin Concentration on Cutinase Activity

**Fig. 13:**
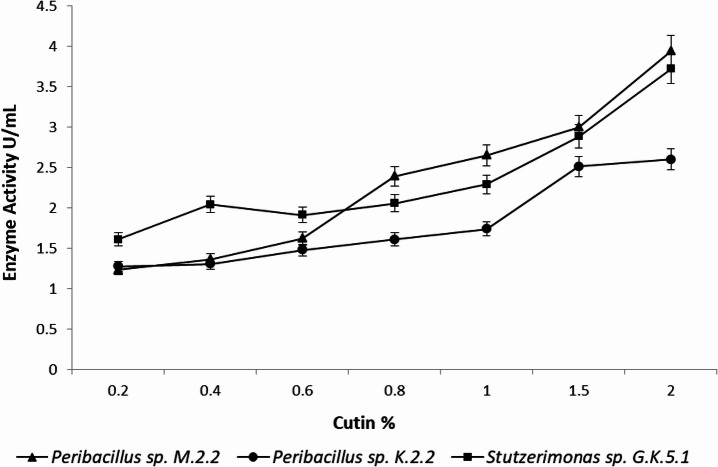
Effect of cutin concentration on cutinase activity of the bacterial isolates. Statistical analyses were performed using one-way ANOVA followed by Tukey’s test for normally distributed data and Kruskal–Wallis followed by Dunn’s test for non-normally distributed data (*P* < 0.05; *n* = 3)

To evaluate the effect of cutin concentration on cutinase activity, tomato-derived cutin was incorporated into MSM medium at concentrations ranging from 0.2% to 2.0% (w/v). Each medium was inoculated with 2% (v/v) of bacterial culture and incubated at 30 °C with shaking at 150 rpm for 7 days. The highest cutinase activities for each isolate were observed at 2.0% cutin concentration: *Peribacillus sp.* M.2.2: 3.93 U/mL (p = *0.0087*), *Peribacillus sp.* K.2.2: 2.60 U/mL (p = *0.0124*), *Stutzerimonas sp.* G.K.5.1: 3.72 U/mL (p = *0.0006*) (Fig. 13). These findings indicate a positive correlation between substrate availability and enzyme activity, up to 2.0% cutin concentration. Comparable observations have been reported in the literature. For instance, a maximum volumetric cutinase activity of 344 U/mL was reported for *Pseudomonas cepacia* using 1.0% (10 g/L) tomato cutin [45]. Similarly, Liu et al. reported a specific cutinase activity of 6.03 U/µg using 0.2% (2 g/L) cutin with *Botrytis cinerea* [38].

### Optimal Conditions for Growth, Cutinase Activity, and Protein Production

The optimal growth parameters (incubation time, temperature, pH, and cutin concentration) for each isolate were determined through controlled experiments. Corresponding cutinase activities and total protein concentrations were quantified and summarized in Table 2. Among the three isolates, *Stutzerimonas sp.* G.K.5.1 demonstrated the highest cutinase activity of 9.21 ± 0.38 U/mL at 30 °C, pH 9.0, and 2.0% cutin, along with the highest protein concentration (0.27 ± 0.003 mg/mL). In contrast, isolates *Peribacillus sp.* M.2.2 and *Peribacillus sp.* K.2.2 exhibited maximum activities of 4.20 ± 0.11 U/mL and 3.16 ± 0.11 U/mL, respectively, under similar conditions.

**Table 2:** Optimal growth conditions, cutinase-like esterase activities, and total protein content of selected bacterial isolates

Species	Isolated	Incubation time (Day)		Temperature °C		Ph	Cutin %	Enzyme activity U/Ml	Total protein mg/mL
*Peribacillus sp.*	M.2.2	3		30		8	2	4.20 ± 0.11	0.18 ± 0.01
*Peribacillus sp.*	K.2.2	3		30		8	2	3.16 ± 0.11	0.13 ± 0.012
*Stutzerimonas sp.*	G.K.5.1	4		30		9	2	9.21 ± 0.38	0.27 ± 0.003

Data represent mean values of triplicate experiments performed under optimal conditions. Enzyme activity and total protein concentrations were statistically analyzed using one-way ANOVA followed by Tukey’s HSD test (*p* < 0.05)

## Discussion

The present study aimed to isolate and characterize bacterial strains from plastic-contaminated environments for their ability to degrade polycaprolactone (PCL) and to produce extracellular cutinases. Based on 16 S rRNA gene sequence analysis, isolates M.2.2 and K.2.2 were affiliated with the genus *Peribacillus* and were closely related to *Peribacillus frigoritolerans*, forming a well-supported cluster (bootstrap value: 97%). Isolate G.K.5.1 clustered within the genus *Stutzerimonas* and showed the highest similarity (98.76%) to *Stutzerimonas zhaodongensis*. Since 16 S rRNA gene sequence similarity alone may not provide sufficient resolution for definitive species-level assignment among closely related taxa, the isolates were conservatively designated at the genus level. Nevertheless, their close phylogenetic relationships to metabolically versatile species suggest ecological relevance in polyester degradation processes.

Among the 82 isolates screened, three bacterial strains—*Peribacillus sp.* M.2.2, *Peribacillus sp.* K.2.2, and *Stutzerimonas sp.* G.K.5.1—demonstrated the highest hydrolytic activity on PCL-containing media, confirming their capability to depolymerize synthetic polyester substrates. The relatively large degradation zones (22–30 mm, AI up to 8.0) indicated significant extracellular enzymatic activity. Similar observations have been made in previous studies, although with varying efficiencies. For instance, clearance zones were reported for 102 out of 313 isolates in a previous study [46], whereas 87 PCL-degrading fungi were identified from coastal PET waste in another investigation [47]. In comparison, the isolates identified in the present study produced larger degradation zones than those reported for *Phaeophleospora eucalypticola* (13.96 mm) and *Cladosporium spp.* (10.21–13.92 mm). These findings suggest that certain bacterial isolates may exhibit surface-degrading capacities comparable to, or exceeding, some reported fungal systems. Furthermore, all three bacterial isolates also secreted active cutinases in the presence of tomato-derived cutin, with *Stutzerimonas sp.* G.K.5.1 showing the highest activity (9.21 U/mL) under optimized conditions (30 °C, pH 9.0, 2% cutin).

The peak enzyme activities between days 3 and 4 are consistent with earlier studies of microbial cutinases, such as *Fusarium oxysporum* and *Pseudomonas aeruginosa*, which reach maximal secretion within 3–5 days [36, 43]. Moreover, the high cutinase activity observed at 30 °C is consistent with findings from similar studies. For instance, *Fusarium falciforme* was reported to exhibit an activity of 4.82 ± 0.18 U/mL under the same temperature conditions [48]. *Acinetobacter baumannii AU10*, which activity 82.75 U/mL in NBY medium with 0.4% tomato cutin at 37 °C [30]. *Bacillus sp.* KY0701 activity 15.01 ± 1.81 U/mL cutinase with 1.5% apple cutin at 50 °C [34]. Similarly, LC-cutinase has been reported to exhibit optimal enzymatic activity at pH 8.5 and 50 °C [49], while the cutinase obtained from *Stenotrophomonas maltophilia PRS8* demonstrated an activity of 450.58 U/mg and remained stable across a range of temperatures (30–40 °C) and pH levels (8.0–10.0) [39]. However, differences in activity units (U/mL vs. U/mg), substrate composition, and assay conditions limit direct quantitative comparison across studies. Within this methodological context, the cutinase activity observed here can be considered moderate yet biologically relevant.

The present study further supports the growing recognition that bacterial enzymes play a critical role in the biodegradation of synthetic polyesters. While fungal cutinases have been widely studied, bacterial systems—particularly from soil and waste environments—remain underexplored. The current study demonstrates that S. stutzeri secretes functional extracellular cutinase-like enzymes. This finding contributes to the taxonomic diversity of known PCL-degrading bacteria and provides experimental evidence supporting the enzymatic potential of this strain.

The variation in optimal pH among isolates—pH 8.0 for *Peribacillus* strains and pH 9.0 for *Stutzerimonas sp.* G.K.5.1 —likely reflects differences in enzyme conformation or gene regulation. The ability of *Stutzerimonas sp.* G.K.5.1 to maintain high activity in alkaline environments highlights its ecological adaptability and potential industrial relevance, especially in processes that occur under high-pH conditions such as detergent formulations or composting. Its previously reported resilience against chemical and oxidative stress [50] further supports this view, making *Stutzerimonas sp.* G.K.5.1 a suitable candidate for environmentally robust biodegradation systems.

The present study further supports the growing recognition that bacterial enzymes play a critical role in the biodegradation of synthetic polyesters. While fungal cutinases have been widely studied, bacterial systems—particularly those originating from soil and waste environments—remain relatively underexplored. The current study extends this knowledge by demonstrating that *Stutzerimonas sp.* G.K.5.1 actively secretes functional extracellular cutinase and is capable of degrading PCL under mesophilic conditions. Tomato-derived cutin was shown to be an effective, low-cost substrate for enzyme induction. The results indicate a clear, dose-dependent increase in enzyme activity with rising cutin concentrations. This aligns with studies using apple and papaya cutins [34, 48], though tomato cutin—rich in ω-hydroxy fatty acids—appears to provide stronger induction potential [29]. The reproducibility of enzyme activity obtained using locally sourced, single-batch cutin also demonstrates a practical and sustainable approach to biocatalyst production.

The PCL film degradation results further confirmed the biodegradation capability of the isolates. *Stutzerimonas sp.* G.K.5.1 achieved a 10.75% weight loss within 10 days, which is comparable to *Lysinibacillus sp*. (9% after 30 days [51], and *Paecilomyces lilacinus* [52]. The quantitative comparison of these enzymatic and degradative performances with previously reported microbial systems is summarized in Table 3.

**Table 3:** Comparative of PCL degradation and cutinase activity of *Stutzerimonas sp.* G.K.5.1 versus bacterial and fungal

Microorganism/Source	Type	Conditions	Mass Loss (%)/Time	Enzyme Activity	References
*Stutzerimonas sp.* G.K.5.1	Bacteria	30 °C/pH 9.0	10.75% (10 days)	9.21 U/mL	This study
*Paecilomyces lilacinus*	Fungus	30 °C/pH 3.5–4.5	10% (10 days)	-	[52]
*Fusarium falciforme*	Fungus	30 °C/pH 7.0	-	4.82 U/mL	[48]
*Pseudozyma japonica-* Y7-0*9*	Fungus	30 °C	93.3% (15 days)	-	[53]
*Fusarium oxysporum*	Fungus	30 °C/pH 7.0	-	22,7 U/mL	[36, 43]
*Botrytis cinerea*	Fungus	45 °C/pH 9.0	-	6,03 U/µg	[38]
*Amycolatopsis alba* AaCut10	Bacteria	37 °C	85.67% (20 min)	-	[54]
*Lysinibacillus sp.*	Bacteria	30 °C	9.0% (30 days)	-	[51]
*Acinetobacter baumannii* AU10	Bacteria	37 °C/pH 8.0	-	82.75 U/mL	[30]
*Stenotrophomonas maltophilia* PRS8	Bacteria	30–40 °C/pH 8–10	-	450.58 U/mg	[39]
*Bacillus sp. KY0701*	Bacteria	50 °C/pH 7,5	77,4% (10 days)	15,01 U/ml	[34]

The catalytic performance of *Stutzerimonas sp.* G.K.5.1 becomes particularly meaningful when evaluated alongside highly optimized systems. For instance, in comparative studies using purified or recombinant enzymes, systems such as recombinant MtCUT achieved 78.5% PCL degradation within only 36 h, while the marine fungal enzyme AaCut10 demonstrated near-complete degradation within minutes [54, 55]. Furthermore, the recombinant KrCUT from *Kineococcus radiotolerans* has shown significant hydrolytic efficiency [56]. However, it is critical to distinguish between these biotechnologically enhanced systems and natural microbial processes. While recombinant and purified enzymes exhibit accelerated kinetics due to high concentration and optimized catalytic environments, our results were obtained using a wild-type isolate in its natural state, without any genetic modification or costly enzyme purification steps. Taken together, these findings demonstrate that although engineered systems offer higher raw efficiency, *Stutzerimonas sp.* G.K.5.1 exhibits a consistent and steady natural depolymerization ability under mild, mesophilic conditions. This suggests that the isolate provides a more sustainable and economically viable model for in-situ bioremediation, where the use of purified enzymes would be logistically and financially prohibitive.

To further elucidate the nature of the observed 10.75% mass loss, FTIR and SEM analyses were evaluated collectively. PCL exhibits characteristic infrared bands, with peaks at 2944 and 2861 cm⁻¹ for asymmetric and symmetric CH₂ stretching, 1725 cm⁻¹ for C = O, and 1300, 1243, and 1168 cm⁻¹ for C–O, C–C, and C–O–C stretching in the polymer backbone [57]. FTIR spectra demonstrated that the characteristic absorption bands of PCL, particularly the carbonyl stretching at ~ 1720 cm⁻¹ and the C–O vibrations at 1100–1250 cm⁻¹, remained preserved after bacterial treatment, with no detectable peak shifts or new band formation. This finding suggests that the primary polymer backbone did not undergo significant bulk chemical modification. Nevertheless, an increase in the intensity of the carbonyl band at ~ 1720 cm⁻¹ was observed in treated samples, suggesting limited surface-level ester bond hydrolysis and/or degradation of amorphous regions.

Furthermore, SEM micrographs revealed pronounced surface erosion characterized by deep cavities and pitted morphology. The coexistence of significant surface deterioration and minimal bulk chemical alteration confirms that the mass loss induced by *Stutzerimonas sp.* GK51 is predominantly associated with surface-confined degradation rather than diffusion-driven bulk chain scission. Collectively, these findings support a surface erosion–dominated mechanism, where enzymatic activity occurs primarily at the polymer–environment interface, allowing for substantial physical damage while the internal polymer skeleton remains structurally intact.

The molecular weights of cutinases reported from bacterial and fungal sources generally range between 22 and 40.8 kDa [23, 28, 31, 58–60]. However, several studies have described cutinases with higher molecular weights. For instance, the cutinase purified from *Botrytis cinerea* was reported to exhibit a molecular weight of 49.22 kDa based on SDS-PAGE analysis [38]. Similarly, the cutinase from *Thermobifida fusca* KW3 (Tfca) was found to have a molecular weight of 52.4 kDa [61], while a cutinase-like enzyme obtained from *Stenotrophomonas maltophilia* PRS8 showed an approximate molecular weight of 58 kDa [39].

In this context, the ~ 49 kDa protein band observed in *Stutzerimonas sp.* G.K.5.1 is higher than the commonly reported molecular weight range for most microbial cutinases but falls within the spectrum of previously described higher-molecular-weight variants. This difference may reflect structural variations such as additional peptide domains, post-translational modifications, or oligomerization tendencies. Such structural features could potentially influence enzyme stability, substrate specificity, and thermal tolerance, suggesting possible functional adaptations.

Despite its promising results, the study has certain limitations. Enzyme characterization was limited to crude extracts, and purification or structural analyses were not conducted. Thus, the biochemical identity and substrate specificity of the enzymes require further investigation. Additionally, the experiments were restricted to PCL as a model substrate, and future studies should test a broader range of polymers, including PET and PBS. Seasonal variation in the composition of tomato cutin could also influence reproducibility, though this was minimized here by using material from a single local batch.

Future research should focus on isolating and purifying the enzymes from *Stutzerimonas sp.* G.K.5.1 for biochemical and structural characterization, including thermostability, kinetic parameters, and substrate range. Comparative omics studies could elucidate the regulatory pathways governing cutinase expression. Scaling up enzyme production using agro-industrial waste such as tomato pomace could enhance the economic feasibility of bacterial cutinase applications in large-scale bioremediation and recycling systems.

## Conclusions

This study successfully identified bacterial strains capable of degrading PCL and secreting extracellular cutinases, directly addressing the objective of isolating polyester-degrading bacteria from environmental samples. Among these, *Stutzerimonas sp.* G.K.5.1 demonstrated the highest enzymatic activity and PCL degradation efficiency under optimized culture conditions (30 °C, pH 9.0, 2% tomato cutin), fulfilling the aim of determining optimal conditions for enzyme activity. Screening on Tween 20, Tween 80, and PCL confirmed the hydrolytic potential of the isolates, and the use of tomato-derived cutin provided experimental evidence for extracellular cutinase induction, complementing existing genomic data.

These results highlight the underexplored potential of environmental bacterial isolates as sources of alkaline-active enzymes and emphasize their relevance for future plastic biodegradation and enzyme-based recycling strategies. Future studies should focus on protein purification, molecular modeling, and testing across different plastic types to further characterize and enhance the catalytic mechanisms of *Stutzerimonas sp.* G.K.5.1 cutinases for biotechnological applications.

## Supplementary Information

Supplementary Material 1.

Supplementary Material 2.

## Data Availability

Data are contained within the article.
