# Supplementary figures and images for: Enzymatic Degradation of Polycaprolactone by Cutinase-Producing Bacteria Isolated from Plastic-Contaminated Environments
Source: Appl Biochem Biotechnol. 2026 Apr 25;198(8):5607–32. doi: 10.1007/s12010-026-05709-7 (PMC13407619; doi:10.1007/s12010-026-05709-7)

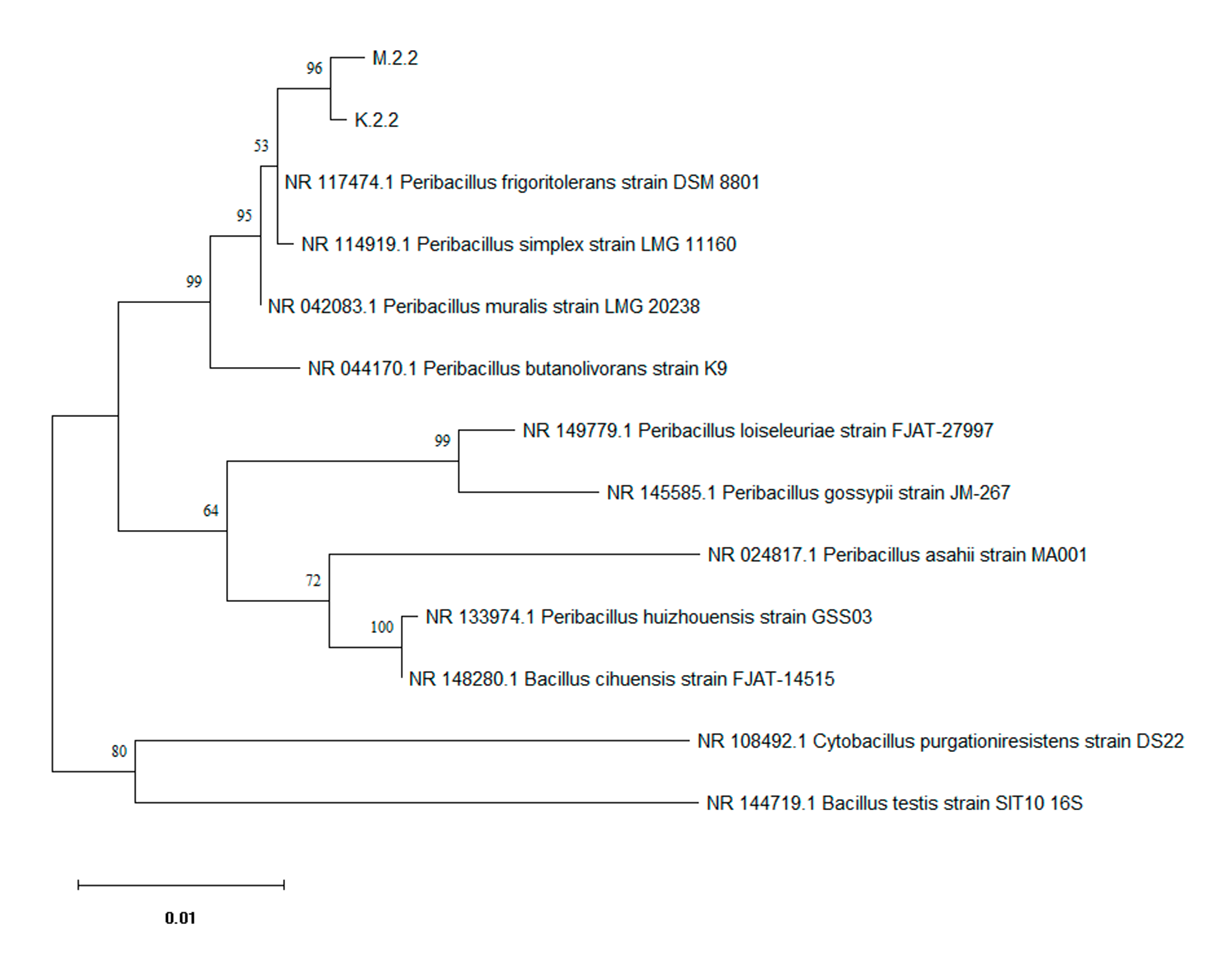

Supplement: Supplementary file 1 — Supplementary Material 1. [file 12010_2026_5709_MOESM1_ESM.png]

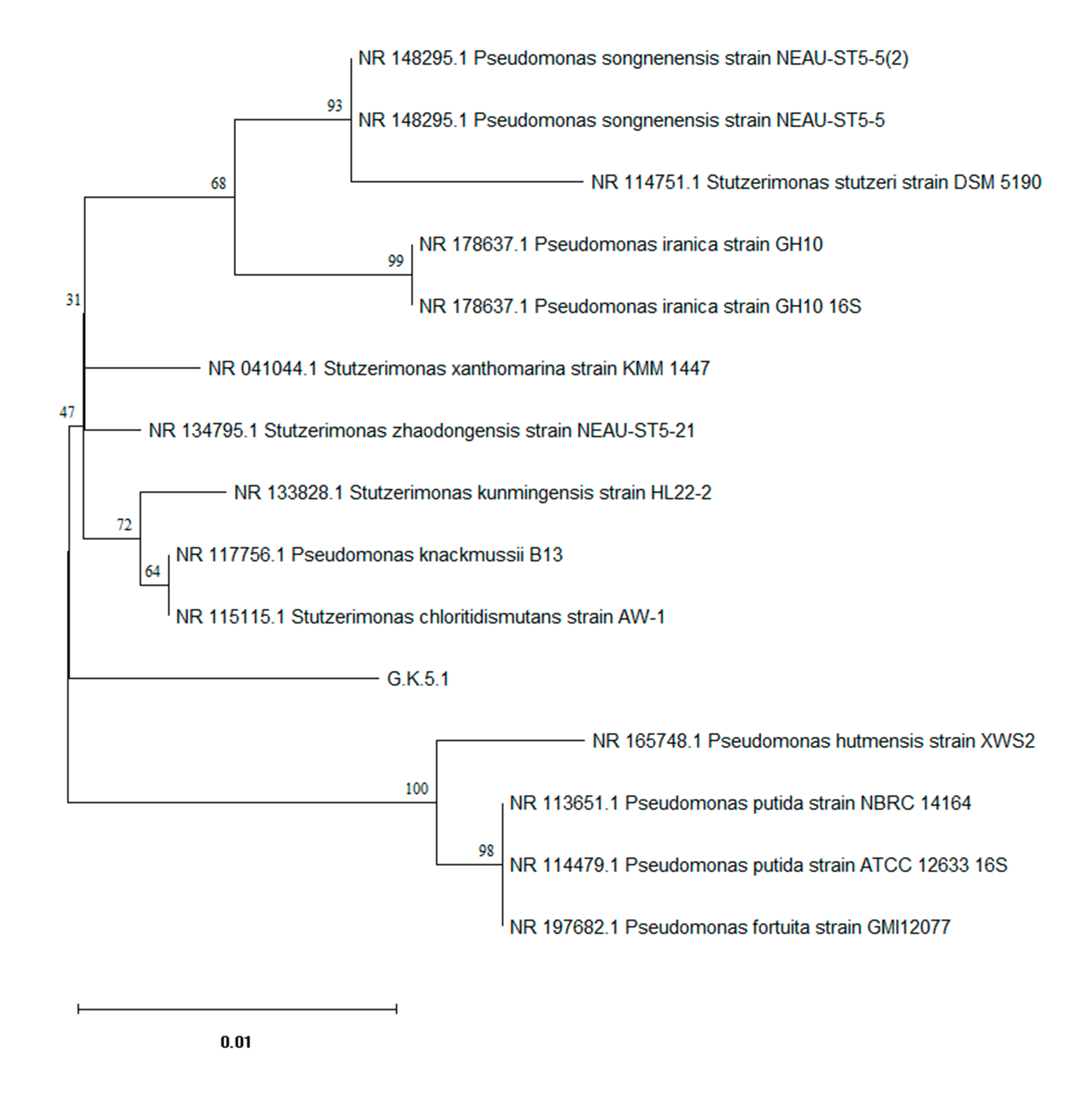

Supplement: Supplementary file 2 — Supplementary Material 2. [file 12010_2026_5709_MOESM2_ESM.png]
